# Investigation of neurotropic arboviruses in wild and domestic animals in Amazon region, 2023-2024

**DOI:** 10.1590/0074-02760250013

**Published:** 2026-03-16

**Authors:** Daniel Jun Hayashi, Bruno Tardelli Diniz Nunes, Sandro Patroca da Silva, Tânia Cristina Alves da Silveira da Cunha, Francisco Amilton dos Santos Paiva, Ivy Tsuya Essashika Prazeres, Alessandra da Conceição Miranda Santos, Landeson Junior Leopoldino Barros, Ana Cláudia da Silva Ribeiro, Felipe Baraldi Sobral, Luiz Mário Fernandes, Taciana Fernandes Souza Barbosa Coelho, Valéria Lima Carvalho, Ana Cecilia Ribeiro Cruz, Daniele Barbosa de Almeida Medeiros, Livia Medeiros Neves Casseb

**Affiliations:** 1 Instituto Evandro Chagas, Ananindeua, PA, Brasil; 2 Agência de Defesa Agropecuária do Estado do Pará, Rondon do Pará, PA, Brasil; 3 Laboratório Central do Estado do Pará, Manaus, AM, Brasil; 4 Fundação Oswaldo Cruz-Fiocruz, Instituto Aggeu Magalhães, Recife, PE, Brasil

**Keywords:** Encephalitis virus, arboviruses, Alphavirus, Flavivirus, Orthoflavivirus, Brazil

## Abstract

**BACKGROUND:**

Arboviruses represent a potential threat to global public health due to their ability to infect various vertebrate hosts and vectors, as well as their adaptability to diverse ecosystems, allowing them to expand geographically across continents.

**OBJECTIVE:**

The present study aimed to describe the molecular epidemiology of encephalitic arboviruses, including *Venezuelan equine encephalitis virus* (VEEV), *Eastern equine encephalitis virus* (EEEV), *Western equine encephalitis virus* (WEEV), *West Nile virus* (WNV), *Saint Louis encephalitis virus* (SLEV), *Toscana virus* (TOSV), and *Rift Valley fever virus* (RVFV) in nervous tissue samples from domestic and wild animals from the Northern region of Brazil, between February 2023 and June 2024.

**METHODS:**

Samples negative for rabies virus were analysed by reverse transcription real-time polymerase chain reaction (RT-qPCR) targeting *Alphavirus*, *Orthoflavivirus* and *Phlebovirus* species.

Positive samples were subjected to viral isolation in cell culture and whole-genome sequencing using next-generation sequencing.

Phylogenetic and molecular clock analyses were performed to characterise viral lineages and evolutionary relationships.

**FINDINGS:**

Two samples tested positive for arboviruses by RT-qPCR: one SLEV sample from the state of Amazonas, which showed a low viral load, preventing virus isolation and sequencing, and one *Madariaga virus* (MADV) sample from the state of Pará, which could be isolated and sequenced.

The isolated agent belongs to lineage III of EEEV, showing most similarity to strains from Guyana and Argentina.

**MAIN CONCLUSIONS:**

The present study detected two arboviruses in animals, suggesting its circulation in the study area.

## INTRODUTION

Arboviruses are viruses that have part of their replicative cycle in arthropods, which serve as a link to transmit these pathogens to animals as well as humans through haematophagy, resulting in an impact on public health and socio-economic conditions. Among the diversity of arboviruses, viruses belonging to the genera *Alphavirus*, *Orthobunyavirus*, *Nairovirus*, *Phlebovirus*, *Orbivirus*, *Vesiculovirus*, and *Thogotovirus* stand out, exhibiting a high mutation rate, which leads to greater adaptability in replicating in different vectors and hosts.^1^

In the group of emerging and re-emerging infectious diseases, arboviruses are considered significant challenges for public health. The detection of the emergence of these viruses is often complicated due to the occurrence of different arboviruses in the same geographical area and successive outbreaks involving different genera with similar symptoms.^2^

Encephalitic agents from the *Alphavirus*, *Orthoflavivirus*, *Phleboviruses* and *Orthobunyavirus* genera have demonstrated global expansion and public health impact in recent years.^3^ ^,^ ^4^ ^,^ ^5^ ^,^ ^6^ Various domestic and wild animals are susceptible to encephalitic arboviruses, which facilitate the maintenance and spread of viral agents, causing economic and social losses. Nevertheless, despite the low probability of severe clinical outcomes, there is a significant number of severe encephalitis cases that may lead to the patient's death or result in long-term sequelae. Thus, there is a significant impact on public health, as well as human losses.^4^ ^,^ ^6^

The effectiveness of surveillance depends on rapid diagnosis in areas where a competent vector exists, and the population is susceptible. Early recognition of local transmission, followed by rapid and effective vector control and other public health measures, is essential to prevent outbreaks. In this context, alongside the rabies surveillance network in animals, the present work aims to expand the monitoring of the distribution of encephalitic agents.

## MATERIALS AND METHODS

*Ethical aspects* - The project was approved by the Animal Use Ethics Committee (CEUA) of the Evandro Chagas Institute (IEC/SVSA/MS) with registration number 23/2023 and followed the guidelines of Resolution No. 51, dated May 19, 2021, of the National Council for the Control of Animal Experimentation - Ministry of Science and Technology (CONCEA-MCT). Regarding biosafety, the professionals involved used individual and collective protective equipment appropriate for the analyses conducted, following the institution's standard operating procedures. The waste generated was disposed of in accordance with the IEC Waste Management Programme.

*Samples* - The laboratory for rabies diagnosis from Department of Arboviruses and Haemorrhagic Fever of the IEC (SEARB/IEC), serving as a macro-regional reference to detection of *Lyssavirus rabies* (RABV), and received samples from wild and domestic animals suspected of rabies infection from various states in the North (Amazon rain forest) and Northeast regions. These samples were sent by municipal and state health departments, as well as state agricultural defence agencies.

A total of 331 nervous tissue samples from wild and domestic animals were analysed for the detection of RABV using biological test (BT) in mice^7^ and direct immunofluorescence (DIF).^8^ The samples included: non-human primate (n = 102), sloth (n = 25), agouti (n = 6), opossum (n = 6), anteater (n = 3), marsupial (n = 2), porcupine (n = 1), coati (n = 1), armadillo (n = 1), paca (n = 1), and capybara (n = 1); as well as bovine (n = 49), feline (n = 26), canine (n = 95), equine (n = 8), donkey (n = 2), swine (n = 1) and ovine (n = 1). These samples were collected from February 2023 to June 2024, from states of Alagoas (n = 66), Amazonas (n = 144), Amapá (n = 2), Pará (n = 64), Rondônia (n = 28), Roraima (n = 3), Rio Grande do Norte (n = 1) and Tocantins (n = 23).

Samples that tested negative for RABV were further analysed using reverse transcription real-time polymerase chain reaction (RT-qPCR) to detect the genomes of encephalitic arboviruses, including: Alphaviruses - *Alphavirus Venezuelan* (Venezuelan equine encephalitis virus - VEEV), *Alphavirus eastern* (Eastern equine encephalitis virus - EEEV), and *Alphavirus western* (Western equine encephalitis virus - WEEV); Orthoflaviviruses - *Orthoflavivirus nilense* (West Nile virus - WNV) and *Orthoflavivirus louisense* (Saint Louis encephalitis virus - SLEV); and Phleboviruses - *Phlebovirus toscanaense* (Toscana virus - TOSV) and *Phlebovirus riftense* (Rift Valley fever virus - RVFV). Positive samples were selected for viral isolation in cell culture, followed by next generation sequencing (NGS) for genome characterisation.

*RNA extraction* - A tissue fragment of approximately 20 mg was placed in a 2 mL microtube with a 5 mm steel bead and 1 mL of lysis buffer from the Maxwell® 16 Tissue LEV Total RNA Purification Kit (Promega, USA, 2015). The samples were homogenised using a TissueLyser II at a frequency of 25 Hz for 2 min. The supernatant was then subjected to RNA extraction using the same purification kit, following the manufacturer's instructions on the automated Maxwell® 16 Instrument. (Promega, Wisconsin, USA) MS2 phage RNA was used as an exogenous internal control to validate the extraction process.^9^

*RT-qPCR* - The primers and probes used in this project targeted WEEV,^10^ WNV,^11^ SLEV^12^ and RVFV,^13^ as well as EEEV, VEEV and TOSV, which were developed by the molecular biology laboratory of SEARB/IEC [Table I and Supplementary data (Table I)]. The probes and primers for MS2 were purchased from integrated DNA technologies (IDT).

**TABLE I:** Sequences of the primers and probes that were used in this research

Virus	Target	Sequence
MADV/ EEEV	EEEV ALL F	5'-GTGGAACAACAACCARARCTTYTT-3'
	EEEV ALL R	5'-GGGATCCCCACCTTGTTCG-3'
	EEEV ALL Probe	5'-FAM-ATCACYGCT/ZEN/GTGTGYTCGTAYGCTGC-IBFQ-3'
WEEV^10^	WEEV 8220F	5'-AGGGATACCCCGAAGGTT-3'
	WEEV 8322R	5'-GTGAATAGCACACGGGTGGTT-3'
	WEEV 8274 Probe	5'- HEX-CTTTCGAAT/ZEN/GTCACGTTCCCATGCG-IBFQ-3'
VEEV	VEEV NSP1 41 F	5'- AAGTTCACGTTGAYATCGAG-3'
	VEEV NSP1 166R	5'-GCGAAAACGCUCURGC-3'
	VEEV NSP1 103 Probe	5'- Cy5-CAGTTTGAG/TAO/GTAGAAGCCAAGCAGGT-3IAbRQSp-3'
WNV^11^	WNRTF	5'-CGGAAGTYGRGTAKACGGTGCTG-3'
	WNRTR	5'-CGGTWYTGAGGGCTTACRTGG-3'
	WNV MGB Probe	5'-FAM-WCCCCAGGWGGACTG-3MGBEq-3'
SLEV^12^	SLEV PrM 834F	5'-GAAAACTGGGTTCTGCGCA-3'
	SLEV PrM 905R	5'-GTTGCTGCCTAGCATCCATCC-3'
	SLEV PrM 857p	5'-HEX-TGGATATGC/ZEN/CCTAGTTGCGCTGGC-IBFQ-3'
RVFV^13^	RVFV F	5'-TGCCACGAGTYAGAGCCA-3'
	RVFV R	5'-GTGGGTCCGAGAGTYTGC-3'
	RVFV Probe	5'-FAM-TCCTTCTCC/ZEN/CAGTCAGCCCCAC-IBFQ-3'
TOSV	TOSVS 1631 F	5'-AAGGTTCAGCACAATCATCATCTTCAC-3'
	TOSVS 1808 R	5'-ATTGCTCTTGCTTTTCTTGATGAG-3'
	TOSVS 1773c Probe	5'-HEX-TGGGACCAT/ZEN/CAATGCATGGGTGAATGA GTTTGCTTA-IBFQ-3'
MS2^9^	MS2 F	5´-CATAAGTTAGATGGCCGTCTGT-3´
	MS2 R	5'- TAGAGACGACAACCATGCCAAAC -3'
	MS2 S Probe	5'-FAM-TCCAGACAA/ZEN/CGTGCAACATATCGCGACGTATCGTGATATGG-IBFQ-3'

EEEV: Eastern equine encephalitis virus; MADV: Madariaga virus; RVFV: Rift Valley fever virus; SLEV: Saint Louis encephalitis virus; TOSV: Toscana virus; VEEV: Venezuelan equine encephalitis virus; WEEV: Western equine encephalitis virus; WNV: West Nile virus.

The duplex SLEV/WNV and TOSV/RVFV protocols, as well as the triplex EEEV/WEEV/VEEV RT-qPCR, which were optimised and standardised at the Biomol SEARB laboratory, using the AriaMx Real-Time PCR instrument. The reaction was set up in a total volume of 25 µL using the Superscript III One-step qRT-PCR Kit (Invitrogen) protocol with the following controls: exogenous internal control, negative extraction control, positive control [MADV (KJ469641), WEEV (not deposited in public genome database), VEEV (AF075253), WNV (MH643887), SLEV (EF158048), RVFV (NC014395) and TOSV (NC006318)] and No Template Control.

For the duplex protocol, the reaction mix included 12.5 µL of RT-qPCR Master Mix (2x) with ROX™ Reference Dye, 1 µL of forward/reverse primers for SLEV (25 µM) and WNV (25 µM), 1 µL of forward/reverse primers for TOSV (50 µM) and RVFV (50 µM), 0.5 µL of probe for SLEV (10 µM) and WNV (10 µM), 0.5 µL of probe for TOSV (10 µM) or RVFV (12,5 µM), 0.5 µL of RT-Mix 40x, and the volume was adjusted to 20 µL with RNase-free H_2_O, along with 5 µL of RNA.

For the triplex protocol, the reaction mix included 12.5 µL of RT-qPCR Master Mix (2x) with ROX™ Reference Dye, 1 µL of forward/reverse primers for EEEV (50 µM), 1 µL of forward/reverse primers for WEEV (25 µM), 1 µL of forward/reverse primers for VEEV (35 µM), 0.5 µL of probe for EEEV (10 µM), 0.5 µL of probe for WEEV (12,5 µM), 0.5 µL of probe for VEEV (12,5 µM), 0.5 µL of RT-Mix 40x, and the volume was adjusted to 20 µL with RNase-free H_2_O, along with 5 µL of RNA.

*Viral isolation* - Homogenised nervous tissues of the animals positive in the RT-qPCR were inoculated, proportion 1:10, in C6/36 and VERO cell culture, using Leibovitz's L-15 and 199 media, respectively. Viral adsorption was performed for 1 h at a temperature of 28ºC (C6/36) or 37ºC (VERO), with gentle homogenisation every 15 min. The infected cells were observed daily for seven days, using inverted optical microscope (Zeissm Axiovert S100) to detect possible cytopathic effects (CPE) in the cell monolayer. If no CPE was observed, the infected cell culture was collected on the 7th day post-inoculation (DPI), and submitted to RT-qPCR and indirect immunofluorescence (IIF) to confirm viral replication. The IFIs were performed using hyperimmune mouse ascitic fluids to group-reactive: group A (*Alphavirus*), group B (*Orthoflavivirus*), and *Oropouchevirus* (*Orthobunyavirus*).^14^

*Next generation sequencing and bioinformatic analysis* - After RNA extraction, quantification was performed using the Qubit RNA HS Assay Kit and Qubit 4.0, followed by cDNA preparation using the SuperScript^TM^ VILO^TM^MasterMix and NEBNext® Second Strand Synthesis Module kits, and purification with the PureLink® PCR Purification Kit. The synthesised cDNA was quantified using the Qubit Assay DNA HS Kit on the Qubit 4.0 and used for genomic library preparation with the Nextera XT Kit. The sequencing was performed on the NextSeq platform 550 (Illumina, Inc.) using the paired-end methodology with the NextSeq High Output Kit (300 Cycles) protocol.

First, the quality of the reads generated during the sequencing step was evaluated using the Fastp program (https://github.com/OpenGene/fastp), with the removal of short reads (less than 50 nt), adapter fragments, and reads with indeterminate bases (reads with N). Next, the SortMeRNA v.4.3 program^15^ was used to remove ribosomal RNA (rRNA). The files generated during the processing step were used for de novo assembly using the SPAdes v3.13.1^16^ and MEGAHIT v1.2.9^17^ programs, using k-mer values of 21, 33, 55, and 77; and 21, 31, 41, 51, 61, 71, 81, 91, and 99, respectively. The generated contigs were analysed using the DIAMOND program^18^ with BLASTX, based on the non-redundant viral protein database (nr), and visualised in the Megan program, using an e-value filter of 1e-10. The contigs that showed similarity with viral sequences of interest were visualised in the Geneious Prime program (https://www.geneious.com/).

*Phylogenetic analysis and identity matrix* - Phylogenetic inference was performed using nucleotide sequences of viral variants from the NCBI database (http://www.ncbi.nlm.nih.gov), focusing on structural protein coding regions. Multiple sequence alignment (MSA) was done with MAFFT v7 program,^19^ and the alignment was manually corrected in Geneious Prime version 2025.0.2. Additionally, a nucleotide and amino acid identity matrix was generated. The presence of recombination among the aligned sequences was assessed using the GARD method available on the Datamonkey website (https://www.datamonkey.org/gard), with the following parameters: run mode set to "faster"; genetic code set to "Universal"; two rate classes; and three types of site-to-site rate variation models (None, General Discrete, and Beta-Gamma).

The best nucleotide substitution model was identified, and the phylogenetic signal was evaluated using TREE-PUZZLE^20^ which plots each tree on a triangular surface. Fully resolved trees fall into the corners, while unresolved quartets are located in the centre of the triangle. If more than 15% of quartets are unresolved, the data are considered inappropriate.

Phylogenetic trees were constructed using the maximum likelihood (ML) method^21^ in IQ-TREE2 v2.2.5,^22^ with 1000 bootstrap replicates.^23^ Phylogeny visualisation was performed with FigTree v1.4.4 (https://github.com/rambaut/figtree/releases/tag/v1.4.4), using midpoint rooting. A scalable vector graphics (SVG) file was generated for editing with Inkscape (https://inkscape.org/release/inkscape-1.3.2/).

*Molecular clock* - To investigate the temporal dynamics of different MADV strains, a molecular clock analysis was performed on the open reading frames (ORFs) from the viral sequences with complete genome sequence and available collection time data. MSA was performed with MAFFT v7.^19^ Subsequently, TempEst v1.5.3^24^ was employed to estimate the temporal signal within the dataset by examining the correlation between sampling dates and genetic distances to assess the temporal resolution. This analysis was informed by a phylogenetic tree generated with IQ-TREE2 v2.3.6.^22^

Each of the six model configurations was set up using BEAUTi v10.5.0^25^ and tested through three independent runs. The configurations comprised two clock models: a strict clock and an uncorrelated relaxed clock.^26^ Each clock model was paired with one of three coalescent models: a constant size coalescent,^27^ a Bayesian SkyGrid^27^ and a Gaussian Markov random field (GMRF) Bayesian SkyRide.^27^

Each model configuration was executed in BEAST v10.5.0^25^ to conduct the molecular clock analysis. Chain lengths were extended to achieve effective sample size (ESS) values above 200. The results were assessed with Tracer v1.7.2, focusing on convergence and overall model performance. The marginal likelihood estimation using path sampling (PS) and stepping-stone sampling (SS) were applied to identify the most suitable model for the dataset.^28^

The selected model was then processed with TreeAnnotator v10.5.0^25^ to summarise and annotate the phylogenetic trees. Finally, the phylogenetic analysis results were visualised in FigTree (http://tree.bio.ed.ac.uk/software/figtree/), facilitating interpretation and presentation.

## RESULTS

*Virus molecular assay* - Out of a total of 331 samples received, seven tested positives for rabies, with one from Amazonas, five from Pará, and one from Tocantins. Therefore, a total of 324 samples were analysed, of which two (0.61%) tested positive by RT-qPCR. One sample was identified as SLEV, and the other as *Alphavirus madariaga* (Madariaga virus- MADV).

**Fig. 1:**
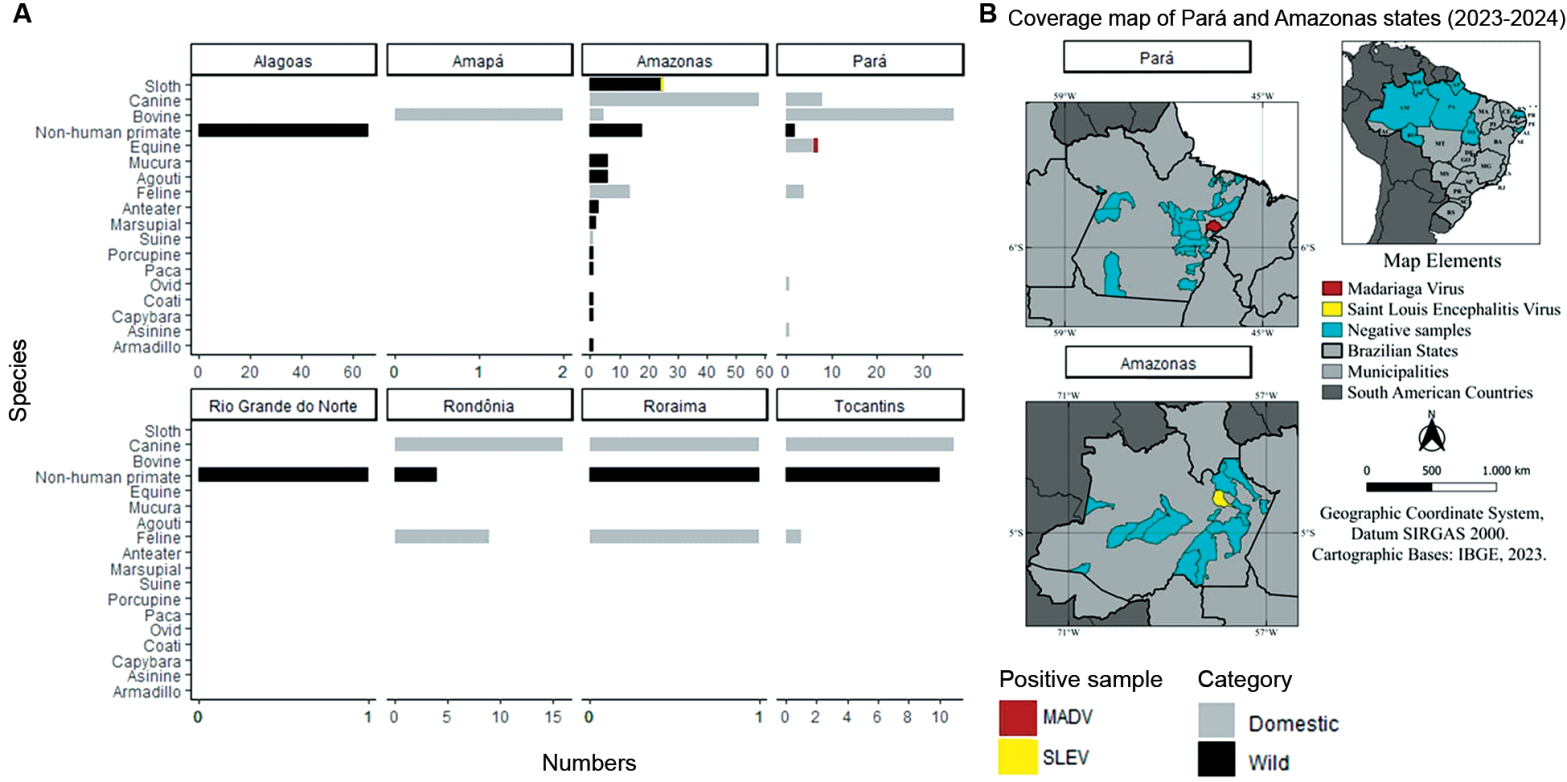
number of samples investigated with the location of the collected area of Madariaga virus (MADV) and Saint Louis encephalitis virus (SLEV). (A) Graph showing the number of samples tested distributed by wild and domestic/agricultural animals across various states in Brazil, totalling 324 samples. (B) Coverage map of the states of Pará and Amazonas, highlighting municipalities where MADV and SLEV were detected, shown in red and yellow, respectively.

The SLEV positive sample (ID: ED930/BeAn884582) originated from the state of Amazonas, where 143 samples were evaluated. This positive sample was collected in the municipality of Manaus from *Choloepus didactylus* (Linnaeus's two-toed sloth) received on November 17, 2023 (Fig. 1). The animal was discovered dead in its natural habitat. The virus isolation was attempted, however, the low viral load [cycle threshold (Ct) = 32] prevented isolation and also rendered genome sequencing.

The MADV positive (ID: UN10477/BeAN889345) sample was detected in the state of Pará, where 59 samples were assessed. The sample was collected in the municipality of Rondon do Pará from an *Equus ferus* (horse), received on May 14, 2024 (Fig. 1). The animal was a male aged 18 months, presented clinical signs including behavioural changes, altered posture/locomotion, staggering, and circling behaviour over a period of three days. The sample showed a Ct value of 22, indicating high viral load. Virus isolation was successfully achieved, and the presence of MADV was confirmed by IFA and RT-qPCR, followed by successful sequencing.

From this infected cell culture, the complete coding DNA sequence (CDS) and a partial non-coding regions (5' and 3') of MADV genome were obtained, corresponding to 99.9% of full genome, showing 11,594nt in length. The mean coverage was 24× across the genome. The sequence was deposited in Genbank, number PQ558655.

The typical alphavirus genomic organisation is preserved, with two main ORFs observed, encoding nonstructural and structural proteins, respectively. The genome follows the order: 5' cap - (nsP1-nsP2-nsP3-nsP4) - (CP-E2-E1) - poly(A)-3'. Both BlastX analysis and BlastN as well as identity matrix showed the highest similarity [ammino acid (aa): 99.78%; nucleotide (nt): 98.61%; e-value = 0.0] with a MADV sequence isolated from *Equus ferus caballus* in Guyana in 1962 (GenBank: KJ469578).

**Fig. 2:**
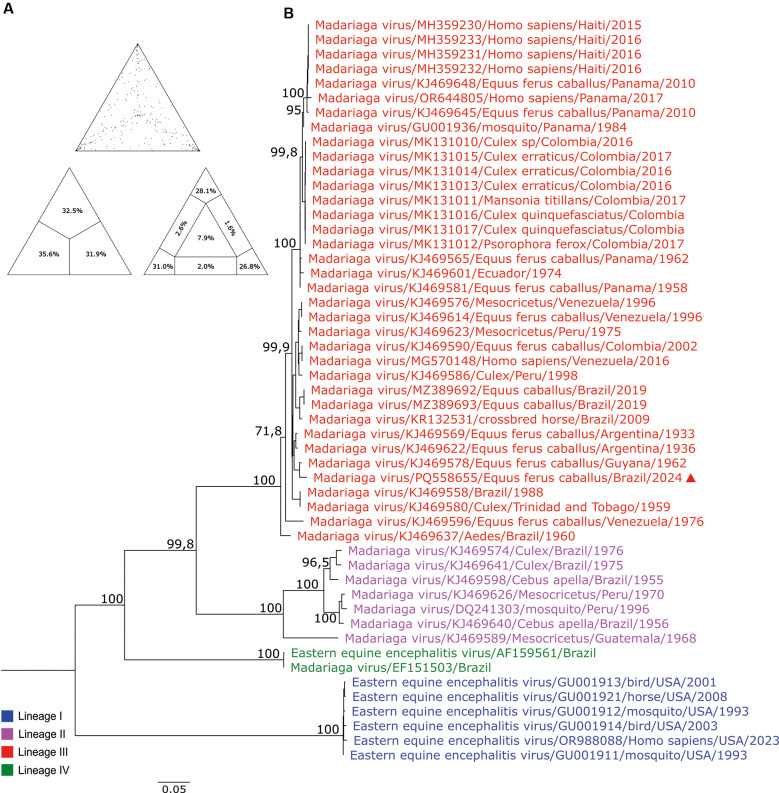
phylogenetic tree of different virus strains belonging to the Eastern equine encephalitis virus (EEEV) complex. (A) Likelihood mapping of Madariaga virus (MADV) dataset. Measurement of phylogenetic signal shows 85,9% of resolved quartets; 6,2% partly resolved quartets and 7,9% of unresolved quartets (B) The maximum likelihood (ML) method was used based on the complete nucleotide sequences of the structural protein, employing the GTR+F+I+G4 matrix as the best nucleotide substitution model. Known lineages are labelled in different colours. Samples identified in this study are highlighted with a red triangle. The numbers at each major node of the tree represent bootstrap values in percentage (1000 replicates). The scale bar corresponds to nucleotide divergence per site between sequence.

The ML tree was built with 51 sequences [Supplementary data (Table II)] of ORFs that encode structural proteins from EEEV lineage.^29^ ^,^ ^30^ No evidence of recombination was detected and the percentage of dots in unresolved quartets was 7.9% (Fig. 2A). The UN10477 sequence clustered within a monophyletic clade of the MADV lineage III, closely related to the equine isolated from Guyana (KJ469578/Guyana/1962, Boostrap: 100%), and other MADV isolates from Argentina collected between 1933-1936 (Boostrap: 92.7%) (Fig. 2B).

The nucleotide identity matrix [Supplementary data (Figure)] matched the organisation of the phylogenetic tree (Fig. 2), with UN10477 showing greater similarity to MADV lineage III.

**Fig. 3:**
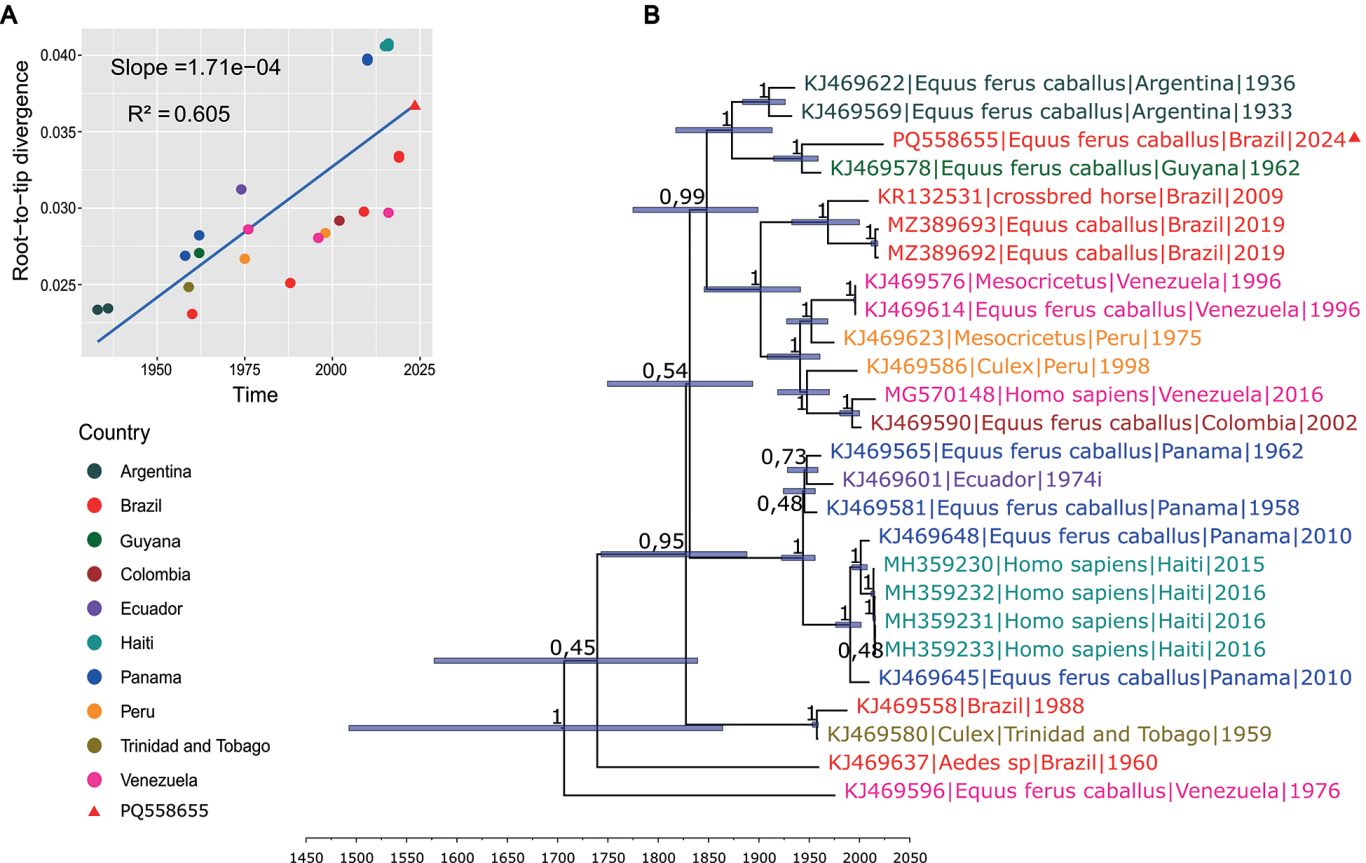
temporal dynamics of the different strains of the Madariaga virus (MADV). (A) Root-to-tip analysis using linear regression between the collection date and divergence distance. (B) Phylogenetic tree analysis based on time-scaled Bayesian inference. Each node represents the posterior probability support, and the blue bar correspond to 95% highest posterior density (HPD) interval that represent the uncertainty in the time to most recent common ancestor (TMRCA) for those clades. The divergence time for each clade can be visually inferred by projecting a vertical line from the node to the time-scaled X-axis. All the sequences from lineage III were not selected because some of them do not have data available regarding the collection date and only complete genome sequence were included.

The temporal signal had a Slope value of 1.71e-04 (p-value = 2.88e-06) and an R-squared of 0.605 (Fig. 3A) having positive correlation in genetic divergence and sampling time for phylogenetic molecular clock analysis. The best-fitting molecular clock model was the uncorrelated relaxed clock paired with the Bayesian SkyGrid. This model had an estimated evolutionary rate of 1,25e-04 substitutions per site per year (95% HPD interval: 6,54e-05 to 1,92e-04) with PS and SS values of -28422.38 and -28422.20, respectively (Table II). The phylogenetic tree estimated the mean year value for the node of the clade containing PQ558655, KJ469578, KJ469569, and KJ469622 to be 1869 with 95% HPD interval of 1817,9 to 1912,9 (Fig. 3B).

**TABLE II:** Six models configurations for molecular clock analysis

Clock type	Tree prior	Length of chain	Path sampling	Stepping stone sampling
Strict clock	Constant size	5,00E+07	-28448,8718	-28448,5614
Strict clock	Constant size	5,00E+07	-28448,6177	-28448,4488
Strict clock	Constant size	5,00E+07	-28449,6215	-28449,4039
Strict clock	Bayesian skygrid	5,00E+07	-28447,6246	-28447,6141
Strict clock	Bayesian skygrid	5,00E+07	-28456,7644	-28456,6223
Strict clock	Bayesian skygrid	5,00E+07	-28448,0490	-28448,4519
Strict clock	GMRF Bayesian skyride	5,00E+07	-28449,8180	-28449,7006
Strict clock	GMRF Bayesian skyride	5,00E+07	-28450,2185	-28449,9547
Strict clock	GMRF Bayesian skyride	5,00E+07	-28449,7259	-28449,5267
Uncorrelated relaxed clock	Constant size	2,00E+08	-28424,9145	-28424,5184
Uncorrelated relaxed clock	Constant size	2,00E+08	-28424,5897	-28424,4118
Uncorrelated relaxed clock	Constant size	2,00E+08	-28424,9650	-28424,5068
Uncorrelated relaxed clock	Bayesian skygrid	2,00E+08	-28422,8338	-28422,5312
Uncorrelated relaxed clock	Bayesian skygrid	2,00E+08	-28426,1797	-28426,5661
Uncorrelated relaxed clock	Bayesian skygrid	2,00E+08	-28422,3869	-28422,2041
Uncorrelated relaxed clock	GMRF Bayesian skyride	2,00E+08	-28424,0788	-28423,5740
Uncorrelated relaxed clock	GMRF Bayesian skyride	2,00E+08	-28424,6073	-28424,4704
Uncorrelated relaxed clock	GMRF Bayesian skyride	2,00E+08	-28424,1785	-28423,9712

GMRF: Gaussian Markov random field.

**Fig. 4:**
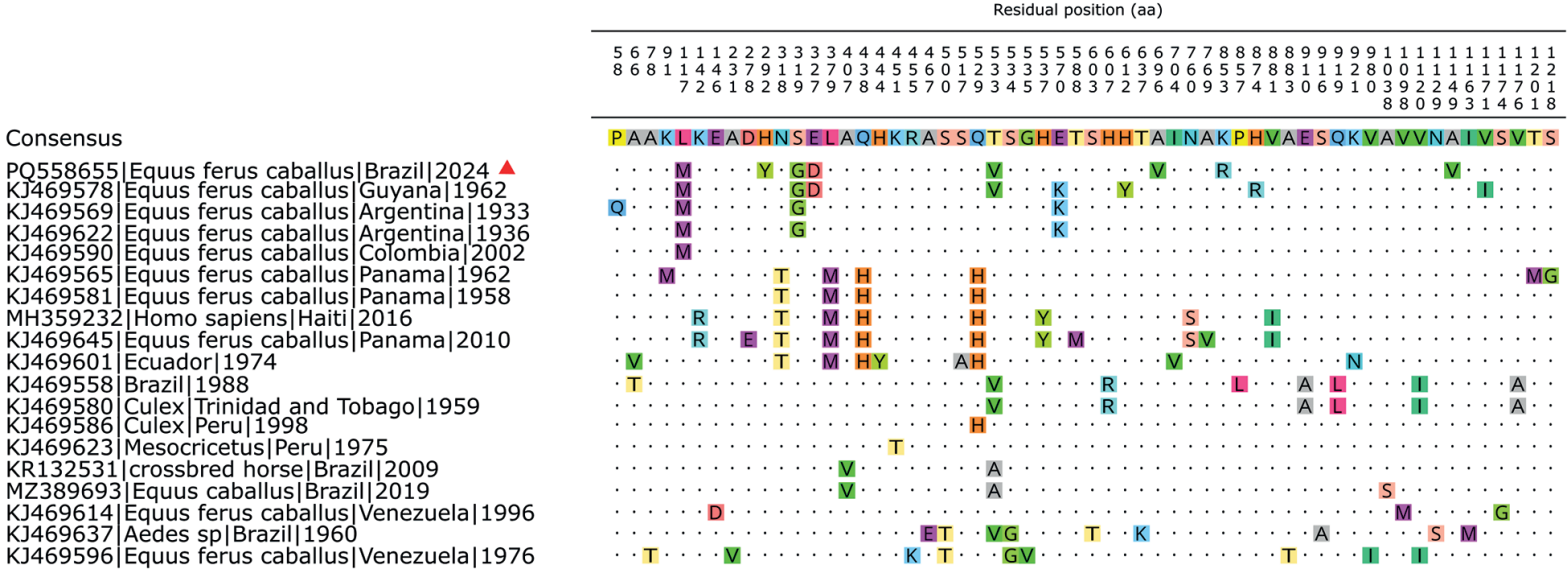
mutation region of amino acids with respective residual position. The red triangle symbol identifies the taxon of the present work. The consensus sequence is constructed from each most frequent residual position.

By examining the amino acid alignment of the structural protein ORF for MADV lineage III (Fig. 4), three clade-defining substitutions were identified. The clade formed by PQ558655, KJ469578, KJ469569, and KJ469622 consistently exhibits the substitutions L177M, S319G, and E570K, which represent changes from the consensus residues to those present in this lineage III subgroup. These shared substitutions distinguish this clade from the remaining lineage III sequences, and the mutation pattern is fully concordant with the phylogenetic clustering observed in Figs 2-3.

## DISCUSSION

The low viral load and RNA degradation commonly observed in nervous tissue from wild animals found dead in tropical environments represent major limitations for molecular detection in surveillance studies. These factors reduce the sensitivity of RT-qPCR and hinder successful viral isolation, particularly when carcasses are exposed to heat and humidity for extended periods. However, serological test results may be affected by cross-reactivity, and only molecular testing or viral isolation can confirm infection with greater specificity.^31^ For this reason, the negative result of the samples analysed in this study does not indicate that the animals had no contact with encephalitic arboviruses.

The identification of SLEV in a sloth located in the municipality of Manaus may support previous findings,^32^ which detected total antibodies for SLEV in young military personnel in the same municipality. However, because the detection in this study relied solely on RT-qPCR with low viral load, it is not possible to determine the genotype or infer the geographic origin of this SLEV strain.

The MADV identified in the equine was found to be the cause of death of the animal, similar to what was reported with isolates from equines in the states of Ceará, Paraíba, and Pará, regarding behavioural changes, ataxia, circling, and a clinical course of three-five days.^29^ ^,^ ^33^ ^,^ ^34^ However, other study reports additional clinical signs such as blindness, depression, severe ataxia, head pressing, salivation, tongue paralysis, and a clinical course lasting up to 15 days.^35^ Compared to EEEV of lineage I, MADV is less lethal,^29^ with a minimal number of human cases during epizootic periods.^36^ However, neurological impairment should not be overlooked, both in humans and animals.

The virus detected in this study belongs to MADV lineage III, a lineage distributed across Central and South America and associated with outbreaks in humans and equines. Human cases linked to lineage III have been documented in Haiti and Panama, where MADV was isolated from serum of children with acute febrile illness during 2015-2016^37^ and from equine nervous tissue during the human outbreak in 2010.^38^ It has been suggested that children are the most vulnerable group to MADV infection, as in Panama, in 2010, children under 10 years old showed signs of acute febrile illness and seizures with epilepsy.^38^ In 2017, Panama reported a fatal human case confirmed by viral isolation.^39^

The MADV from the present study shows high similarity to the virus identified in Argentina, being the first outbreak case in the country in equines in 1930, in Buenos Aires,^36^ indicating high sequence conservation within lineage III, with a mean evolutionary rate of 1.25e-04 which is more similar to the 1.2e-04 previously reported^30^ and slightly lower than 1.8e-04 estimated for EEEV,^40^ as well as its persistence in South America. Other outbreak cases have been reported in Argentina, such as in 1981 in horses in the province of Santiago del Estero,^41^ and in 1988 in Castelli, in the province of Chaco.^36^

Previous molecular clock analyses suggest that MADV lineage III originated in Brazil and subsequently subdivided into two clades (IIIa and IIIb). The IIIa clade spread from Brazil to Argentina, Peru, and Trinidad, whereas the IIIb clade spread from Panama to Haiti.^30^ This phylogenetic framework supports the placement of the isolate described in the present study within the broader evolutionary diversification of lineage III.

The vertebrate hosts involved in the maintenance of MADV are not yet fully established; however, detections in Brazil have included *Didelphis marsupialis*, wild boars, and tapirs.^42^ ^,^ ^43^ ^,^ ^44^ In Pará, the IEC isolated 64 MADV/EEEV from 33 chicks, 13 primates, five birds, four reptiles, three rodents, three equines, two marsupial and one bat,^45^ indicating continuous viral circulation in the state and suggesting that this circulation is maintained by vertebrate hosts with limited mobility.^46^ Arboviruses associated with such hosts would have less shared molecular epidemiological patterns, exhibiting independent and local evolution.^47^ However, the present study identified a MADV with greater similarity to those from Argentina in 1933 and 1936, compared to other isolates in Brazil from 2009 and 2019. The vector that has shown the greatest prominence in the life cycle of MADV is *Culex* of the subgenus *Melanoconion*.^46^ In Pará, MADV was isolated from *Culex* (*M.*) *taeniopus*,^48^ which may be the main vector in the region, as it is in Panama.^49^ In Peru, of the mosquitoes isolated, *Cx.* (*Mel.*) *pedroi* was the most prominent.^50^ In Argentina, the mosquito *Culex* (*Culex*) spp. is the most prevalent compared to the subgenus *Melanoconion*.^36^ ^,^ ^46^ Together, these findings highlight the importance of continued molecular surveillance of MADV lineage III, particularly in regions with ongoing viral circulation, to improve understanding of its evolutionary dynamics and epidemiological impact.

*In conclusion* - Our findings underscore the value of molecular surveillance for detecting and characterising encephalitic arboviruses and highlight the need for continued monitoring to better understand the circulation and evolutionary patterns of MADV lineage III.

## SUPPLEMENTARY MATERIALS

Supplementary material

## Data Availability

The viral genome sequence obtained in the present study is available in the NCBI GenBank database under the accession number PQ558655. Other data that support the findings of this study will be shared upon reasonable request author.
