# Supplementary material for: Investigation of neurotropic arboviruses in wild and domestic animals in Amazon region, 2023-2024
Source: Mem Inst Oswaldo Cruz. 2026 Mar 16;121:e250013. doi: 10.1590/0074-02760250013 (PMC12991413; doi:10.1590/0074-02760250013)
Supplement: Supplementary material [file 1678-8060-mioc-121-e250013-s1.pdf]

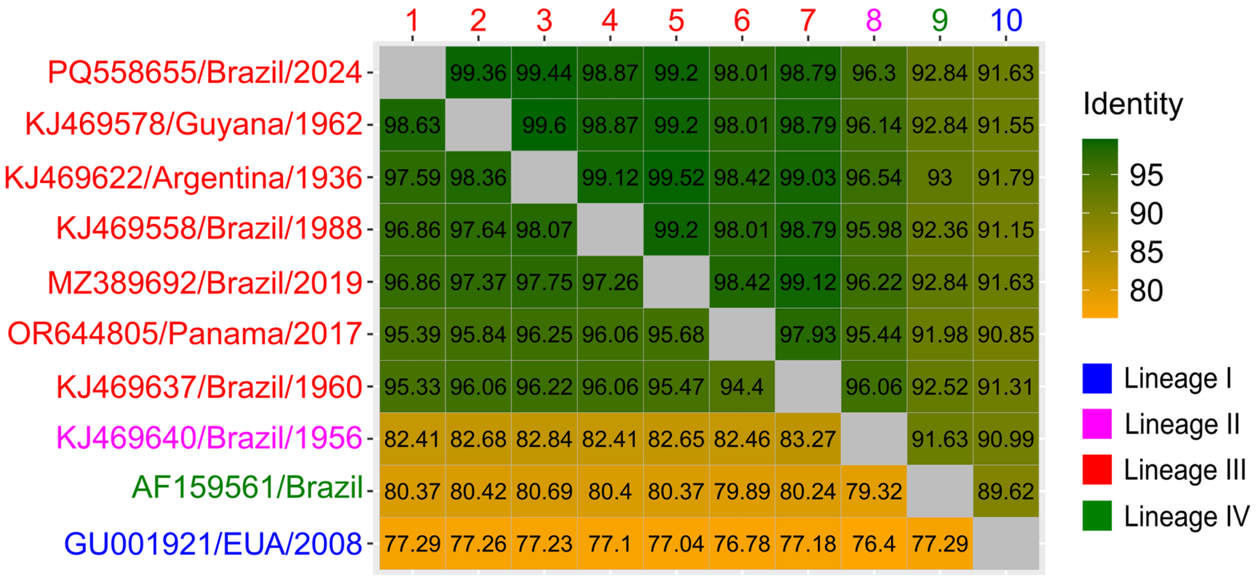

Heatmap of divergence matrix of nucleotides and amino acids. The matrix positioned in the lower left corner of the map represents the nucleotide identity. The matrix positioned in the upper right corner of the map represents the amino acid identity.

TABLE I

Reverse transcription real-time polymerase chain reaction (RT-qPCR) protocols used in the present study

Reference	Target	Limit of detection	Positive predictive value	Negative predictive value	EFF%
SEARB	MADV/EEEV	Be published	Be published	Be published	Be published
Kang et al.[10]	WEEV	Two copies	Not done	Not done	92.29
SEARB	VEEV	Be published	Be published	Be published	Be published
Vázquez et al.[11]	WNV	Five copies	Not done	Not done	86.407
Lanciotti and Kerst[12]	SLEV	0.15 PFU	Not done	Not done	83.71
Weidmann et al.[13]	RVFV	10^2 copies	Not done	Not done	75
SEARB	TOSV	Be published	Be published	Be published	Be published

EEEV: *Eastern equine encephalitis virus*; EFF: efficiency; MADV: *Madariaga virus*; RVFV: *Rift Valley fever virus*; SEARB: Department of Arboviruses and Haemorrhagic Fever; SLEV: *Saint Louis encephalitis virus*; TOSV: *Toscana virus*; VEEV: *Venezuelan equine encephalitis virus*; WEEV: *Western equine encephalitis virus*; WNV: *West Nile virus*.

TABLE II

Metadata of the sequences used for comparison

ID	Sequence Lenght (bp)	Organism	Host	geo_loc_name	Collection date	Isolate	Lineage
GU001911.1	3729	*Eastern equine encephalitis virus*	Mosquito	USA: Florida	1993	Not found	1
GU001912.1	3729	*Eastern equine encephalitis virus*	Mosquito	USA: Florida	1993	Not found	1
GU001913.1	3729	*Eastern equine encephalitis virus*	Bird	USA: Georgia	2001	Not found	1
GU001914.1	3729	*Eastern equine encephalitis virus*	Bird	USA: Texas	2003	Not found	1
GU001921.1	3729	*Eastern equine encephalitis virus*	Horse	USA: Tennessee	2008	Not found	1
OR988088.1	11681	*Eastern equine encephalitis virus*	*Homo sapiens*	USA: Alabama	Aug-2023	R135167b	1
KJ469574.1	11601	*Madariaga virus*	*Culex*	Brazil	1976	Not found	2
KJ469589.1	11582	*Madariaga virus*	*Mesocricetus*	Guatemala	1968	Not found	2
KJ469598.1	11620	*Madariaga virus*	*Cebus apella*	Brazil	1955	Not found	2
KJ469626.1	11626	*Madariaga virus*	*Mesocricetus*	Peru	1970	Not found	2
KJ469640.1	11624	*Madariaga virus*	*Cebus apella*	Brazil	1956	Not found	2
KJ469641.1	11602	*Madariaga virus*	*Culex*	Brazil	1975	Not found	2
DQ241303.1	11604	*Madariaga virus*	Mosquito	Peru	1996	Not found	2
GU001936.1	3729	*Madariaga virus*	Mosquito	Panama	1984	Not found	3
KJ469558.1	11602	*Madariaga virus*	Not found	Brazil	1988	Not found	3
KJ469565.1	11607	*Madariaga virus*	*Equus ferus caballus*	Panama	1962	Not found	3
KJ469569.1	11608	*Madariaga virus*	*Equus ferus caballus*	Argentina	1933	Not found	3
KJ469576.1	11608	*Madariaga virus*	*Mesocricetus*	Venezuela	1996	Not found	3
KJ469578.1	11609	*Madariaga virus*	*Equus ferus caballus*	British Guiana	1962	Not found	3
KJ469580.1	11605	*Madariaga virus*	*Culex*	Trinidad and Tobago: Trinidad	1959	Not found	3
KJ469581.1	11607	*Madariaga virus*	*Equus ferus caballus*	Panama	1958	Not found	3
KJ469586.1	11608	*Madariaga virus*	*Culex*	Peru	1998	Not found	3
KJ469590.1	11587	*Madariaga virus*	*Equus ferus caballus*	Colombia	2002	Not found	3
KJ469596.1	11609	*Madariaga virus*	*Equus ferus caballus*	Venezuela	1976	Not found	3
KJ469601.1	11607	*Madariaga virus*	Not found	Ecuador	1974	Not found	3
KJ469614.1	11608	*Madariaga virus*	*Equus ferus caballus*	Venezuela	1996	Not found	3
KJ469622.1	11473	*Madariaga virus*	*Equus ferus caballus*	Argentina	1936	Not found	3
KJ469623.1	11608	*Madariaga virus*	*Mesocricetus*	Peru	1975	Not found	3
KJ469637.1	11600	*Madariaga virus*	*Aedes*	Brazil	1960	Not found	3
KJ469645.1	11495	*Madariaga virus*	*Equus ferus caballus*	Panama	2010	Not found	3
KJ469648.1	11498	*Madariaga virus*	*Equus ferus caballus*	Panama	2010	Not found	3
KR132531.1	11534	*Madariaga virus*	Crossbred horse	Brazil	2009	MADV/crossbred horse/ BR/PR/01/2009	3
MG570148.1	11561	*Madariaga virus*	*Homo sapiens*	Venezuela	06/jun/16	Not found	3
MH359230.1	11546	*Madariaga virus*	*Homo sapiens*	Haiti	20-Apr-2015	Not found	3
MH359231.1	11546	*Madariaga virus*	*Homo sapiens*	Haiti	19-Feb-2016	Not found	3
MH359232.1	11546	*Madariaga virus*	*Homo sapiens*	Haiti	23-May-2016	Not found	3
MH359233.1	11546	*Madariaga virus*	*Homo sapiens*	Haiti	25-May-2016	Not found	3
MK131010.1	3698	*Madariaga virus*	*Culex* sp.	Colombia	12-Dec-2016	P1	3
MK131011.1	3698	*Madariaga virus*	*Mansonia titillans*	Colombia	17-Feb-2017	P2	3
MK131012.1	3698	*Madariaga virus*	*Psorophora ferox*	Colombia	17-Feb-2017	P3	3
MK131013.1	3698	*Madariaga virus*	*Culex erraticus*	Colombia	15-Dec-2016	P4	3
MK131014.1	3698	*Madariaga virus*	*Culex erraticus*	Colombia	15-Dec-2016	P5	3
MK131015.1	3698	*Madariaga virus*	*Culex erraticus*	Colombia	15/jan/17	P6	3
MK131016.1	3698	*Madariaga virus*	*Culex quinquefasciatus*	Colombia	Not found	P7	3
MK131017.1	3698	*Madariaga virus*	*Culex quinquefasciatus*	Colombia	Not found	P8	3
MZ389692.1	11605	*Madariaga virus*	*Equus caballus*	Brazil	May-2019	Not found	3
MZ389693.1	11605	*Madariaga virus*	*Equus caballus*	Brazil	May-2019	Not found	3
OR644805.1	11613	*Madariaga virus*	*Homo sapiens*	Panama: Darien	2017	Not found	3
PQ558655	11594	*Madariaga virus*	*Equus ferus caballus*	Brazil	14-May-2024	UN10477/AN889345	3
AF159561.1	3799	*Eastern equine encephalitis virus*	Not found	Brazil	Not found	BR85-436087	4
EF151503.1	11661	*Madariaga virus*	Not found	Brazil	Not found	Not found	4
